# Antibiotic resistance mechanisms and global resistance patterns of *Pseudomonas aeruginosa* in microbial keratitis

**DOI:** 10.1007/s10096-026-05484-7

**Published:** 2026-03-23

**Authors:** Tanzina Akter, Shiful Islam, Abrar Maswood Haider, Kaniz Fatema, Fiona Stapleton, Mark Willcox

**Affiliations:** 1 School of Optometry and Vision Science, Faculty of Medicine and Health, University of New South Wales (UNSW), Sydney, NSW 2052 Australia; 2 Microbial Biotechnology Division, National Institute of Biotechnology (NIB), Dhaka, 1349 Bangladesh; 3 Department of Biotechnology, Faculty of Natural Science, Norwegian University of Science and Technology, Trondheim, 7034 Norway; 4 Department of Microbiology, Primeasia University, Banani, Dhaka 1213 Bangladesh

**Keywords:** *Pseudomonas aeruginosa*, Microbial keratitis, Antibiotic resistance, Resistance mechanisms

## Abstract

**Background:**

Microbial keratitis (MK) is a rapid and devastating infection that can result reduced vision, with lack of treatment potentially resulting in stromal necrosis and even permanent vision loss. *Pseudomonas aeruginosa* is a common cause of MK and its rise in antibiotic resistance has made it increasingly difficult to treat.

**Purpose:**

This review aims to provide a better understanding of the resistance mechanisms of *P. aeruginosa* and highlights major adaptations to combat fluoroquinolones, aminoglycosides, β-lactams and polymyxin antibiotics commonly used in MK, and addresses the global resistance profiles of *P. aeruginosa* keratitis.

**Method:**

A narrative review was conducted using PubMed, Scopus, Web of Science, MEDLINE, and Google Scholar. Search terms included “*Pseudomonas aeruginosa*”, “microbial keratitis”, “antibiotic resistance”, antibiotic class-specific resistance terms, “surveillance studies”, and “regional resistance patterns” to consolidate current information of the various intrinsic, acquired and adaptive resistance mechanisms of *P. aeruginosa* conferred across fluoroquinolones, aminoglycosides, β-lactams and polymyxin along with resistance profile of keratitis isolates across continents.

**Results:**

*P. aeruginosa* displays complex resistance mechanisms, including intrinsic efflux systems, reduced porin permeability, enzymatic drug inactivation, horizontal gene transfer, and target-site mutations, contributing to MDR in MK. Resistance patterns vary markedly by region, with higher resistance to fluoroquinolones, cephalosporins, and aminoglycosides reported in Asia, while Europe and North America showed lower rates. Australian isolates demonstrate heterogeneous resistance, retaining susceptibility to aminoglycosides.

**Conclusion:**

Future studies comparing resistance mechanisms and data of *P. aeruginosa* across regions will be essential to identify geographical variations, inform region-specific surveillance, guide targeted therapies to improve interventions of MK.

## Introduction

*Pseudomonas aeruginosa* is highly adaptive opportunistic pathogen that causes microbial keratitis (MK), a particularly severe ocular infection of the cornea [1–3]. MK occurs primarily after corneal trauma, ocular surface injuries or during contact-lens use [4]. The infection leads to corneal inflammation, pain, photophobia, increased tear production, visual blurring, purulent ocular discharge and ulceration [4]. It may result in stromal necrosis, scarring, and ultimately blindness or irreversible vision loss, when treatment is compromised [5]. Treatment of *P. aeruginosa* keratitis relies on intensive topical antibiotic therapy [6]. High concentrations of fluoroquinolones such as ciprofloxacin or ofloxacin 0.3% (w/v) are commonly administered as monotherapy, typically initiated with hourly dosing that is reduced as the infection improves [4, 7, 8]. In severe or antibiotic-resistant cases, fortified antibiotic combinations consisting of cephalosporins (e.g., ceftazidime) and aminoglycosides (e.g., tobramycin or gentamicin) are utilized [4, 9].

*P. aeruginosa* has multiple intrinsic antibiotic resistance mechanisms, including efflux pumps, reduced outer membrane permeability, and production of antibiotic-inactivating enzymes [10, 11]. *P. aeruginosa* can also acquire further resistance determinants through horizontal gene transfer, and the associated genes can produce β-lactamases, aminoglycoside-modifying enzymes, as well as mutating its genes which is commonly associated with fluoroquinolone resistance [12, 13]. The convergence of these mechanisms drives the emergence of multidrug-resistant (MDR) strains that pose significant therapeutic challenges [12]. Alarmingly, over 40% of *P. aeruginosa* isolates from corneal samples can be MDR, defined as acquired resistance to at least one agent in three or more antimicrobial classes [14]. Besides, *P. aeruginosa* exhibits an epidemic population structure characterized by frequent recombination events, which contribute to the emergence of highly successful epidemic clones [15]. A recent outbreak in the USA caused by a particular sequence type (ST1203) of *P. aeruginosa* associated with lubricating eye drops has resulted in 81 identified cases of severe MK with vision loss (14 patients), enucleation of the eye (4 patients), and death (4 patients) [16]. This *P. aeruginosa* clone (VIM-GES-CRPA) is resistant to virtually all antibiotics or combination of antibiotics [16].

Although antimicrobial resistance in *P. aeruginosa* has been extensively studied in different infections, there is still a lack of a comprehensive synthesis that focuses on resistance mechanisms and resistance data of keratitis isolates across the world. This review summarized the antibiotic resistance mechanisms and resistance profile of keratitis *P. aeruginosa* isolates in different continents.

## Antibiotic resistance mechanisms

**Fig. 1:**
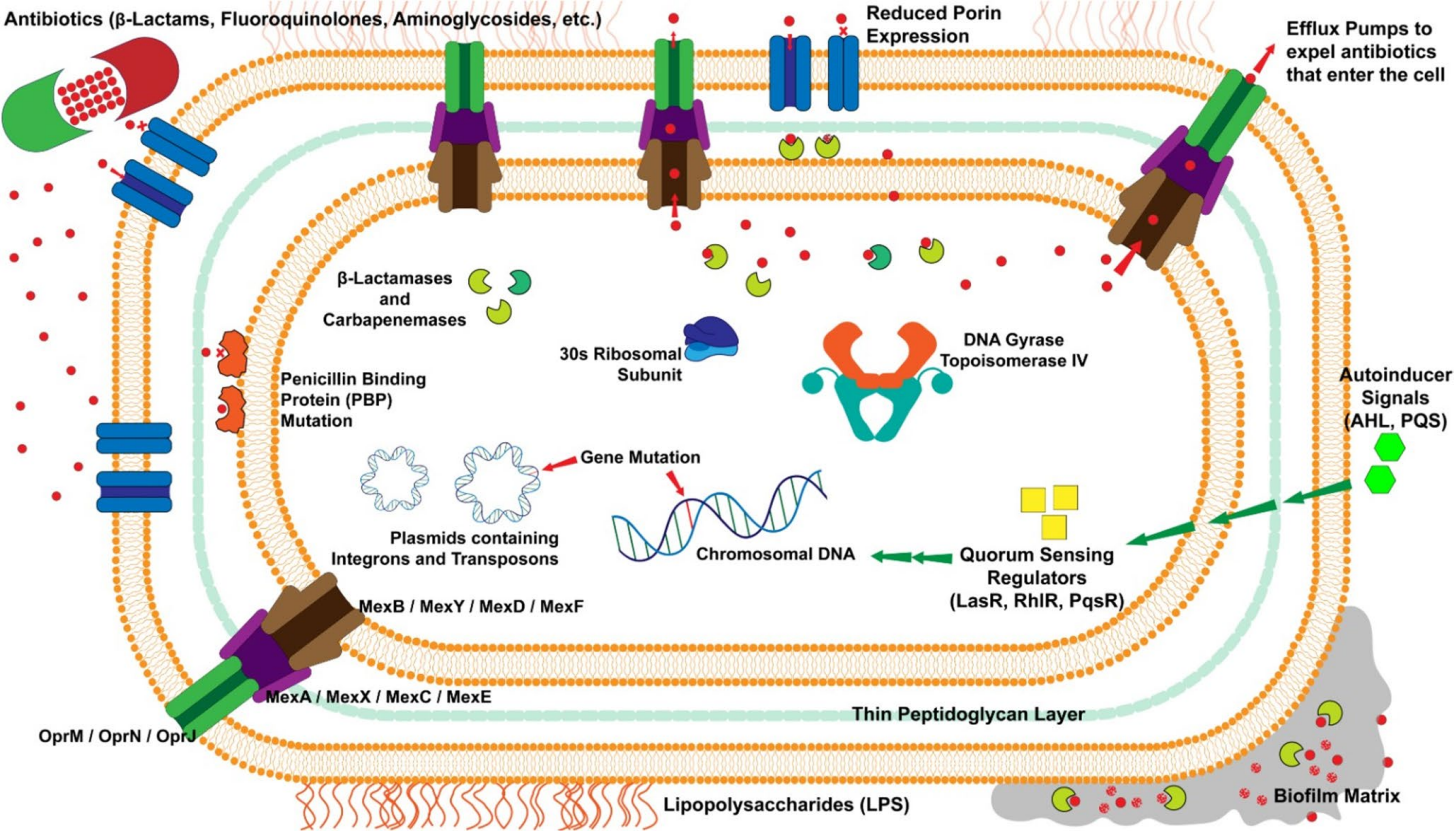
Intrinsic, adaptive and acquired resistance mechanisms of *P. aeruginosa* in MK

*P. aeruginosa* employs a wide variety of innate (intrinsic), adaptive and acquired resistance characteristics some of which are ubiquitous to many antibiotics while others are specific to antibiotics group (Fig. 1).

### Intrinsic resistance

Intrinsic resistance involves common, naturally occurring, non-mutational characteristics associated with antibiotic resistance [11]. Related genes are typically encoded chromosomally rather than on mobile genetic elements (MGEs) as this demonstrates a fundamental expression in pathogenic populations. In *P. aeruginosa*, these include low outer membrane permeability, efflux complexes and inactivation of antibiotics by AmpC β-lactamase, providing pathogens with an intrinsic basal level of resistance [17].

#### Low outer membrane permeability

*P. aeruginosa*’s outer membrane is composed of an asymmetric phospholipid bilayer, containing lipopolysaccharides (LPS) and β-barrel protein channels which cumulatively create a highly selective barrier. Due to a lack of unspecific porins, antibiotic uptake is exceptionally low in *P. aeruginosa* [18]. Porins can be divided into four classes as either non-specific (OprF), ion-gated (OprC and OprH), substrate specific (OprB, OprD, OprE, OprO and OprP) and efflux porins (OprM, OprN and OprJ) [17]. *P. aeruginosa’s* predominant unspecific porin, OprF, has low antibiotic affinity and is responsible for the nonspecific uptake of saccharides and ions but only around 5% of the pores are open at a given time [19]. Consequently, compared to other bacteria such as *E. coli*, the outer membrane of *P. aeruginosa* is extremely restricted with around 12- to 100-fold lower permeability [19]. Different classes of antibiotic demonstrate specific pathways of cell entry with quinolones and other β-lactams entering through various porin channels including OprB, OprC and OprE, and carbapenems entering through OprD, while polycationic antibiotics including aminoglycosides interact with outer membrane LPS to promote their own uptake [19–21].

#### Efflux pumps

Efflux pumps are frequently present in many different types of bacteria and at varying levels of expression. The efflux system can be categorised into five major families: ATP Binding Cassette (ABC), Major Facilitator Superfamily (MFS), Small Multidrug Resistance (SMR), Multidrug and Toxic Compound Extrusion (MATE), and Resistance Nodulation Division (RND) [22]. The fundamental purpose of efflux pumps is to provide low level resistance to toxic compounds including antibiotics [17].

Efflux pumps from the RND family are the most significant contributors to antibiotic resistance of *P. aeruginosa* [17]. Of the 12 efflux systems of this class, overexpression of MexAB-OprM, MexXY-OprM, MexCD-OprJ, and MexEF-OprN has been directly associated with antimicrobial resistance [23]. RND efflux systems are composed of three sections, a periplasmic membrane fusion protein (PMFP) in the inner membrane, a connecting resistance-nodulation-cell division transporter (RNDt) and a channel-forming outer membrane factor (OMF) in the outer membrane [23]. MexAB-OprM was the first efflux pump discovered in *P. aeruginosa* and confers the broadest range of antibiotics resistance [10]. In many cases, these pumps confer low levels of resistance in conjunction with or development of other more specialised mechanisms [24].

#### Enzyme inactivation

*P. aeruginosa* possesses at least one β-lactamase enzyme as their primary mode of β-lactam resistance [10]. This hydrolytic enzyme disrupts the bonds of the β-lactam ring to deactivate the antibiotic. β-lactamases can be present in 4 different forms (class A-D) and all four classes have been found in *P. aeruginosa* [10]. Chromosomally encoded genes for a β-lactamase class C cephalosporinase (AmpC) and a class D oxacillinase (PoxB) are frequently carried by *P. aeruginosa* [10].

### Acquired resistance

Unlike intrinsic resistance, which is typically stable and chromosomal, acquired resistance occurs either through horizontal gene transfer or mutations to genes [17].

#### Horizontal gene transfer

*P. aeruginosa* PAO1 was the first strain to be sequenced, and has a genome size of 6.3 Mbp [25]. More recent sequencing and comparison of a more diverse range of strains has revealed a relatively conserved genomic structure with interspersed accessory genetic material [25]. A distinction can be made between the highly conserved core genome accounting for 90% of genetic material across strain varieties and the accessory genome which shows inter-strain variability with clusters around specific loci [25]. These variable regions have been termed “regions of genomic plasticity” (RGPs) and can be seen in different strains, typically on MGEs [26]. MGEs including plasmids, transposons, integrons and prophages can be acquired through horizontal gene transfer (HGT) from closely or distantly related bacterial species [17]. HGT can occur through DNA transformation, conjugation or transduction [25]. Transformation is the uptake of DNA from the environment, conjugation is the direct transfer of DNA from one cell to another, and transduction is the transfer of DNA from one bacteria to another via a bacteriophage [27].

#### Mutations

Mutation driven antibiotic resistance involves alterations to genes that may affect β-lactamase production, porin permeability, efflux pump expression or regulation and antibacterial target site structure [17]. These mutations can occur in either the chromosomal or the accessory genome.

#### Enzymatic inactivation of antibiotic

*P. aeruginosa* enzymes are capable of phosphorylating (APH), acetylating (AAC) or adenylating (ANT) aminoglycosides including gentamicin and tobramycin which are in common use in MK [28]. The enzymes AACs are the most common form of enzymatic *P. aeruginosa* resistance to aminoglycosides [28]. All these genes are typically found on transposons and/or integrons, indicating their mode of acquisition and transfer [28]. More recently discovered β-lactamases are frequently acquired through integron, plasmid- or transposon associated genes [10]. These include extended-spectrum β-lactamase (ESBL) enzymes from classes A and D and monobactams, and carbapenemases from classes A, B and D [10].

### Adaptive resistance

*P. aeruginosa* demonstrates a remarkable propensity for environmental adaptation through the combination of the biofilm and quorum sensing systems (QS). The applications of these systems in antibiotic resistance promotes altered responses and gene expression depending on the environmental conditions, allowing for greater levels of antibiotic resistance.

#### Biofilms and quorum sensing

Most bacteria are capable of surface attachment and biofilm formation achieved by self-generated encasing within extracellular polymeric substances (EPS) [29]. In this state, bacteria are able to more successfully evade the host immune system and withstand antibiotic attacks with resistances increasing by up to 1000 times [29]. This enhanced resistance to antibiotics arises from reduced antibiotic penetration, as only the superficial layers are accessible, and reduced metabolic activity [17]. Ciprofloxacin, tetracycline and tobramycin require metabolic activity for their antimicrobial action meaning the inactive subpopulations are resistant [30].

The three quorum sensing pathways in *P. aeruginosa*, Lasl-LasR, Rhll-RhlR and PQS-MvfR contribute to the development of matured biofilms [17, 29]. This process is an adaptive mechanism as communities retain the ability to return to a non-biofilm state with original antibiotic sensitivities restored [19]. Laboratory strains without the capability for quorum sensing do not have the capability for biofilm formation and in turn are more susceptible to antibiotic treatment [31].

Despite quorum sensing typically acting adaptively to enhance antibiotic resistance, it is also a target for antibiotics. Subinhibitory concentrations of some β -lactams such as cefepime and ceftazidime can inhibit quorum sensing and reduce biofilms [32]. Conversely, subinhibitory concentrations of aminoglycosides and other β -lactams including imipenem, induce the formation of biofilms [19, 33]. Other antibiotics such as chloramphenicol and carbenicillin were found to have no effect on biofilm formation [33].

#### Persister and tolerant cells

In certain infections *P. aeruginosa* does not succumb to antimicrobial therapy despite laboratory testing indicating susceptibility [34]. This is attributed to the presence of persister cells [34]. Instead of genetic resistance, persister cells are tolerant to high concentrations of antibiotics due to their metabolic inactivity and lack of active antibiotic targets [19]. They make up around 1% of biofilms and are therefore able to repopulate and reinstate infection [19]. They demonstrate their adaptive resistance as they do not proliferate until the antibiotic has been removed, exemplifying their contribution to chronic infection [19]. The formation of persister cells is enhanced by nutrient deprivation and it is also influenced by communication from the quorum sensing system [19].

In addition to persister cells, *P. aeruginosa* populations can survive transient exposure to high concentrations of antibiotics without acquiring genetic resistance, a phenomenon often referred to as tolerance. This type of survival is particularly evident in biofilm-associated bacteria, where cells are killed more slowly than their planktonic counterparts. Ciprofloxacin exposure of ocular isolates has shown that some bacteria can withstand treatment (tolerant cells) despite being classified as susceptible by standard laboratory tests [35].

## Resistance mechanisms to specific antibiotics groups

### Fluoroquinolones and mechanism of fluoroquinolones resistance

Fluoroquinolone antibiotics act by inhibiting DNA gyrase and topoisomerase IV. They are commonly classified into generations based on their antimicrobial spectrum, with ciprofloxacin, ofloxacin, and norfloxacin representing the second generation, levofloxacin the third generation, and moxifloxacin and gatifloxacin the fourth generation. The DNA gyrase is encoded by genes called *gyrA* and *gyrB* whereas topoisomerase IV is encoded by *parC* and *parE*. Both enzymes are essential for DNA supercoiling, replication, and transcription [36–38]. Fluoroquinolones stabilize DNA breaks and produce bactericidal double-strand breaks during replication by binding the enzyme-DNA complex [39]. Because of this direct action on conserved enzyme targets and excellent cell penetration, fluoroquinolones have been widely used against *P. aeruginosa* [37, 40]. However, resistance to fluoroquinolones has emerged and is frequently caused by (i) site-specific mutations in their target genes [37, 41], (ii) efflux pump overexpression [37, 38, 41], (iii) plasmid-mediated factors [37, 41, 42] and (iv) physiological and structural factors of bacterial cells [17, 43].

#### Target site modifications

Fluoroquinolone resistance develops gradually through a stepwise accumulation of mutations. The quinolone resistance-determining region (QRDR) is located in the amino-terminal domains of GyrA (residues 67–106 in *E. coli* numbering) and ParC (residues 63–102). These regions contain the key binding sites for fluoroquinolones [44–46]. The most frequently observed mutation to *gyrA* is the threonine to isoleucine substitution at codon 83 (Thr83Ile) and this mutation is considered to be the primary step in resistance development. Secondary mutations in topoisomerase IV, particularly the serine to leucine substitution at codon 87 (Ser87Leu) in the *parC* gene, also occur. When selection pressure is maintained, these target-site mutations tend to be the initial step towards clinically significant resistance because they physically decrease drug binding to the enzyme-DNA complex [38, 47–49]. Isolates with both *gyrA* and *parC* genes mutations have shown much higher minimum inhibitory concentrations (MICs) values than those with only one mutation [47, 50–52]. Mutations in the *gyrB* and *parE* genes are less frequent in clinical isolates of *P. aeruginosa* [51, 53]. Fluoroquinolone resistant *P. aeruginosa* MK isolates have these mutations in *gyrA* (Thr83Ile) and *parC* (Ser87Leu) [54–57].

#### Efflux pump overexpression

Members of the RND efflux pumps export fluoroquinolones out of the cell, lowering intracellular drug concentrations and raising MICs [58, 59]. The MexAB-OprM, MexCD-OprJ, MexEF-OprN, and MexXY-OprM are the major efflux pumps involved in fluoroquinolones resistance. Each one is regulated by specific repressor proteins. Mutations in repressor genes of MexAB-OprM efflux pump system, including *mexR*,* nalC*,* nalD* can lead to constitutive overexpression of MexAB-OprM [10]. The MexCD-OprJ and MexEF-OprN systems are regulated by the mutations of *nfxB* and *mexT* repressor genes respectively [60]. MexXY-OprM is controlled by a single known regulator called the *mexZ* repressor gene [10, 61]. Efflux upregulation synergizes with QRDR mutations [62]. *P. aeruginosa* ocular isolates contain mutations in efflux pumps encoding genes *mexX*,* mexT*,* mexD*,* mexM*, and *mexY* [57, 63]. Efflux pump overexpression drives fluoroquinolone resistance in clinical *P. aeruginosa* keratitis isolates [55, 57].

#### Plasmid-mediated quinolone resistance (PMQR)

Plasmid-mediated quinolone resistance (PMQR) first emerged in 1998 with the identification of the *qnr* gene in clinical isolates of *Klebsiella pneumonia*. Three distinct PMQR mechanisms have been characterized to date: Qnr proteins (encoded by genes like *qnrA*,* qnrB*,* qnrC*,* qnrD*,* qnrS*,* qnrVC*, and *qnrE*) which protect the quinolone targets DNA gyrase and topoisomerase IV from inhibition [41, 42, 64]; the variant aminoglycoside acetyltransferase AAC (6’)-Ib-cr, which chemically modifies and inactivates fluoroquinolones such as ciprofloxacin [42, 65]; and plasmid-borne efflux pumps (e.g., *qepA* and *oqxAB*) that actively extrude the antibiotic from bacterial cells [42, 66]. Generally, a low-level of resistance is confer through PMQR elements but they are clinically important because they help bacteria to survive at drug concentration, which promotes the emergence of QRDR mutations and the activation of efflux mechanisms [64, 67]. These resistance genes are often linked to MGEs such as insertion sequences (IS26, ISCR3), transposons, and integrons, helping the genes move easily from one bacterial species to another through horizontal transfer [65, 68].

QRDR mutations along with *qnrVC1* is linked to high-level fluoroquinolone resistance in *P. aeruginosa* isolated from MK, suggesting a synergistic activity between these mechanisms [69]. Several studies have shown that when PMQR coexists with other resistance mechanisms, such as carbapenemase genes, β-lactamase synthesis and efflux pump overexpression, this leads to multidrug-resistant strains that pose significant therapeutic challenges in *P. aeruginosa* [70, 71].

Additionally, a new mechanism of ciprofloxacin resistance in *P. aeruginosa* is represented by the enzyme CrpP (ciprofloxacin resistance protein, plasmid encoded). CrpP is encoded by the *crpP* gene located on the conjugative plasmid pUM505 of *P. aeruginosa* [72]. Unlike chromosomal mechanisms, CrpP directly modifies ciprofloxacin by phosphorylation at the C-3 carboxyl group of ciprofloxacin. As a result of modification, it disrupts the drug’s ability to interact with its cellular targets, DNA gyrase and topoisomerase IV [72–74]. Although, CrpP does not confer high-level resistance, its linkage to the highly transmissible pUM505 plasmid and associated Integrative and Conjugative Elements (ICEs) is particularly concerning [74–77]. In 2018, the *crpP* gene was discovered from the clinical isolate of *P. aeruginosa* in Mexico [72]. Since that discovery, *crpP* gene and its homologs have been detected in many *P. aeruginosa* strains as part of various mobile ICEs in the different locations. *P. aeruginosa* keratitis isolates from Australia and India harbour the *crpP* gene, which was carried in genetic islands containing ICEs [63]. Whilst possession of *crpP* along with QRDR mutations and another fluoroquinolone resistance gene *qnrVC1* was associated with resistance, possessing *crpP* alone was not associated with increased fluoroquinolones resistance [63].

#### Physiological and structural factors

Structural loss or modification of outer membrane porin channels (OprB, OprD, OprE, OprO and OprP) can limit the entry of fluoroquinolone, lowering the intracellular drug concentrations [13, 17]. Another important factor is the creation of biofilms microenvironments which reduce metabolic activity and replication rates. In this situation, fluoroquinolones loss their effectiveness as they alter the DNA replication process [17, 43, 78]. Moreover, biofilm-associated cells often show coordinated stress response, such as SOS induction. These responses increase the rates of mutation and help resistant variants development over time [17, 47, 79]. *P. aeruginosa* isolated from contact lenses can form strong biofilms [80]. Another study showed that a significant proportion of *P. aeruginosa* isolates from ocular infections demonstrated strong biofilm formation [81].

Some *P. aeruginosa* cells transform into persisters and these cells are induced by the quorum-sensing molecules like pyocyanin and 3-OC12-HSL and demonstrate temporary resistance to fluoroquinolones through metabolic dormancy [82]. Additionally, these cells act as a reservoir that re-establishes infection after treatment pressure has subsided since they can survive fluoroquinolones exposure without developing genetic resistance [35, 43, 82, 83].

### Aminoglycosides and mechanism of aminoglycosides resistance

The aminoglycosides represent a class of antibiotics which target the 16 S rRNA (A-site) of the 30 S ribosomal subunit and inhibit protein synthesis. There are four generations of aminoglycosides based on their ability to subvert bacterial resistance [84]. The first generation includes streptomycin, neomycin, and kanamycin. This generation represents the old guard of aminoglycosides which have been around since the early 1940s and were replaced over time due to their high toxicity, narrow spectrum and resistance developed through aminoglycoside modifying enzymes (AMEs) [85]. The second generation consists of only gentamicin which served as a broad-spectrum antibiotic, while the third generation was developed to counter antibiotic-resistance and increasing side-effects such as ototoxicity; nephrotoxicity and neuromuscular blockade [86]. This generation included amikacin, netilmicin, sisomicin, and tobramycin. The fourth generation is isepamicin, developed to overcome resistance from AMEs.

*P. aeruginosa* resists aminoglycoside’s activity via different mechanisms, primarily by inactivating the drug activity using different aminoglycoside-modifying enzymes (AMEs), and 16 S rRNA methyltransferases (RMTases). Other mechanisms include overexpression of efflux pump systems (e.g. MexXY-OprM); reducing membrane permeability to undercut drug absorption; and modifying the drug binding target site of 30 S ribosomal subunit [10, 87].

#### Aminoglycoside modifying enzyme (AME)

Modifications like acetylation by the Aminoglycoside Acetyltransferases (AACs); adenylation by the Aminoglycoside Nucleotidyltransferases (ANTs), and phosphorylation by the Aminoglycoside Phosphotransferases (APH) reduce the ability of aminoglycosides binding to the bacterial 30s ribosome to inhibit protein synthesis [10, 28, 88, 89] .

AACs, have been shown to be effective inactivators of gentamicin, tobramycin, and kanamycin. While acetylation does occur on positions 1-,3-,6′-, and 2′- amino groups, the most notable subfamilies are 3-N-AAC (3) and 6′-N-AAC (6′) [10, 19]. The AAC (3) family contributes to gentamicin resistance while the AAC (6′) contributes to tobramycin, amikacin, and gentamicin resistance [10]. Previous investigations of *P. aeruginosa* reported the presence of *aac* (6′)-Ib9 and *aac* (6′)-Ib10 in keratitis isolates was associated with aminoglycosides resistance [90].

Within ANTs, the most common one is ANT (2′′)-1 which confers resistance to gentamicin, amikacin and tobramycin [10, 19]. The lesser-known ANT (4′)-II confers resistance to tobramycin and amikacin, while ANT (3′) shows resistance to only streptomycin [10]. Previous ocular study has reported multiple variants of the integron-associated aminoglycoside nucleotidyltransferase gene, *aadA* (aminoglycoside adenyl transferase) across several isolates [90].

APHs can inactivate antibiotics such as kanamycin, neomycin, and streptomycin through phosphorylation [10, 19]. In *P. aeruginosa*, APH (3’)-IIb is encoded from chromosomal *aphA* gene conferring resistance to kanamycin; APH (3′)-VI mediates resistance to amikacin; while APH (2′′) is associated with gentamicin and tobramycin resistance [10]. A study on Indian keratitis isolates identified *aph (6)-ld* gene was carried on the Tn5393 transposon within a plasmid, to be linked to streptomycin resistance; while *aph (3*′*)-llb* was detected in all examined isolates [63]. Genomic analysis of ocular isolates of sequence type ST308 identified the presence of *aph (6)-Id*, *aph (3′)-llb* and *aph (3′′)-Ib* highlighting the accumulation of multiple resistance determinants [91]. It has been shown that the presence of the acquired resistance genes *aph (3*′′*)-Ib* and *aph (6)-Id* were strongly correlated to resistance against gentamicin and tobramycin in MK [57]. Another study analyzed whole genome sequencing data of 70 corneal isolates and detected *aph (3*′*)-llb* as the most prevalent APH gene, with a small subset of isolates containing *aph (3*′′*)-lb* and *aph (6)-ld* genes [90]. Collectively, the results demonstrate that while APH-mediated resistance is common across ocular isolates, the repertoire of APH genes varies among individual strains [57, 90].

#### 16S rRNA methylases

The methylation of the 30 S ribosomal subunit via 16 S rRNA methylase modifies the antibiotic target leading to resistance. The 16s rRNA methylases RmtA, RmtD, and ArmA which confer resistance to clinically relevant aminoglycosides [10, 92]. Of which the RmtD2 and RmtB enzymes were reported in ocular isolates of sequence type ST308 with variants also being present in sequence types ST316 and ST235 [91, 93]. Analysis of 39 whole-genome–sequenced data of keratitis isolates revealed that *rmtD2* and *rmtB* resistance genes were possessed by only 10% and 5% of the isolates [57].

#### Efflux pumps, porins and two-components systems

In *P. aeruginosa*, the RND efflux systems play a critical role in antibiotic resistance with the MexXY system being a significant determinant of aminoglycoside resistance [94]. Specific single amino acid substitutions in MexY can cause increased resistance to several antibiotic classes including aminoglycosides [23]. Overexpression of the OprH porin also provides aminoglycoside resistance by preventing antibiotics binding to negatively charged LPS required for cell entry [95]. Loss-of-function mutations in *mexZ*, the TetR-like repressor of the MexXY-OprM efflux system results in efflux pump overexpression. Mutations across the *mexX*,* mexY* genes showed significant resistance to aminoglycosides, including gentamicin and tobramycin in keratitis isolates [57]. This is further compounded by mutations in the ParRS two component regulatory system as well as from mutations in *fusA1* and *armZ* genes which play a role in aminoglycoside resistance in keratitis [57, 96, 97]. Point mutations resulting in single amino acid substitutions, such as Ser170Asn and Leu153Arg on *parR* gene and several mutations on *armZ* gene showed resistance to gentamicin and tobramycin [57].

#### Mobile genetic elements (MGEs)

MGEs, which include transposons, resistance islands, prophages, integrons, and plasmids are responsible for transporting aminoglycoside resistance genes thereby contributing to the rapid spread of aminoglycoside resistance and indeed, multi-drug resistance as well. Acquired AME genes frequently reported in *P. aeruginosa* include *aac (6′)-Ib*,* aac (6′)-ll*,* aac (3′)-ll*,* aph (6)-ld*, and *aadA* variants, while high-level aminoglycoside resistance has also been associated with the 16 S rRNA methyltransferase *armA* [98, 99].

### Beta lactams and mechanism of beta lactams resistance

The beta-lactams represent the class of antibiotics which contain a β-lactam ring and act on the bacteria via penetration through porins; inhibit the peptidoglycan synthesizing enzymes and overall disrupt the cell wall synthesis [100, 101]. This includes the carbapenems (imipenem, meropenem, doripenem), beta-lactamase inhibitors, cephalosporins (ceftazidime, cefepime), penicillins, monobactams (aztreonam) [12]. Carbapenems, often considered as the last line of defence for multi-drug-resistant Gram-negative bacilli [12, 100]. To combat the resistance threat posed by β-lactamase enzymes, β-lactamase inhibitors were developed as adjunctive agents. Although they do not possess intrinsic antibacterial activity, they enhance the activity of β-lactam antibiotics by preventing enzymatic hydrolysis and thereby restoring or extending their antibacterial effect. The combined therapeutic approach of a β-lactams paired with β-lactamase inhibitor ensures that the antibiotic will not be hydrolyzed by the enzymes [102]. The list of inhibitors now includes clavulanate, avibactam, tazobactam and vaborbactam which are being paired with β-lactams such as the combinations of ceftazidime/avibactam, ceftolozane/tazobactam, and imipenem/relebactam [88]. Avibactam is an efficient inhibitor of Class A β-lactamases, which allow ceftazidime-avibactam combinations effective despite the microbe’s developing resistance to ceftolozane-tazobactam (Ahmed et al., 2020). The combination of cefozoxime-avibactam has shown to be effective for broad-spectrum beta-lactamase producing microbes and carbapenem resistant *P. aeruginosa* (CRPA) strains [103].

There are five generations of cephalosporins, stratified based on their spectrum of coverage. Antibiotics from the third, fourth and fifth generations possess coverage for *P. aeruginosa.* These include ceftazidime (3^rd^ generation), cefepime (4^th^ generation), and the 5^th^ generation ceftolozane which is used in combination with β -lactamase inhibitor tazobactam [104]. Cefiderocol, a novel siderophore cephalosporin, has recently emerged as a potential option for the treatment of multidrug- or extensively drug-resistant (XDR) *P. aeruginosa* keratitis [105, 106], particularly following the artificial tears–associated outbreak of XDR *P. aeruginosa* in the United States [107]. In an experimental rabbit keratitis model, topical cefiderocol (50 mg/mL) was well tolerated and demonstrated effective in vitro and in vivo activity against XDR *P. aeruginosa*, with greater efficacy than ciprofloxacin and tobramycin in that setting [105]. Subsequent work showed that its efficacy is influenced by the condition of the corneal epithelium, with reduced corneal concentrations and diminished antibacterial activity when the epithelium remained intact [106]. These findings suggest that cefiderocol may represent a promising future topical therapy for resistant *P. aeruginosa* keratitis, although further clinical studies are needed before routine ophthalmic use can be recommended.

Of the antipseudomonal penicillin’s, there are two broad-spectrum antibiotic classes, carboxypenicillins (e.g. carbenicillin, ticarcillin) and ureidopenicillins (piperacillin, azlocillin), of which piperacillin is also used in combination with β -lactamase inhibitor, tazobactam for treating resistant microbial strains [104]. Within monobactam antibiotics, aztreonam is the only one approved for use in the US, most commonly used to treat *P. aeruginosa* based infections [108].

*P. aeruginosa* relies upon several mechanisms to generate resistance against these antibiotics. It implements β-lactamase enzymes to destroy the antibiotics and mediates dysfunction of its outer membrane porin OprD, which reduces the number of available porins to prevent direct antibiotic entry into the cell [100]. Additionally, the use of efflux pumps to remove antibiotics that do reach within the cell have shown to be vital mechanisms through which *P. aeruginosa* shows resistant to β-lactam antibiotics [109]. Beyond this, biofilm formation helps to strengthen its resistance to the antibiotics [100].

#### Beta lactamases

The β-lactamases are enzymes capable of cleaving the β-lactam ring present within the β-lactam antibiotics, thereby preventing the antibiotics from binding to the penicillin-binding proteins, PBPs, to destabilize the bacterial cell wall by inhibiting cell wall synthesis. β-lactamases can be classified in different categories. The Ambler classification divides them based on amino acid sequence similarity into four molecular classes (Class A, B, C, and D). Functionally, they are grouped into Serine β-lactamases, SBLs (Class A, C, D) which utilize a conserved active-site serine residue to hydrolyse the β-lactam ring, and Metallo-lactamases, MBLs (Class B), which require zinc ions for catalytic activity [102, 110].

Based on substrate profile, the β-lactamases can also be grouped to give Penicillinases, Cephalosporinases (e.g. AmpC), Extended-Spectrum β-Lactamases, ESBL (e.g. hydrolyze Oxacillins, Cefotaximes) and Carbapenemases (e.g. KPC, NDM, IMP, VIM) [102]. In *P. aeruginosa*, the intrinsic β-lactamases (e.g. AmpC) are chromosomally encoded, whereas ESBLs and carbapenemases are typically acquired via mobile genetic elements such as integrons and plasmids [92].

Three different intrinsic β-lactamases were identified in the genome of *P. aeruginosa* strain PAO1, namely the class A PIB-1, class C AmpC and class D PoxB [111]. Horizontal acquisition of some of the other narrow-spectrum and broad-spectrum β-lactamases can result in the formation of high risk clones with global prevalence [92]. The narrow spectrum β-lactamases include Pseudomonas specific enzymes − 1 and 4 (PSE-1, PSE-4) while among the broad-spectrum ones are PER-1 (*Pseudomonas aeruginosa* RNL-1); VEB-1 (Vietnamese extended-spectrum β-lactamase); GES-1 & GES-2 (Guiana extended spectrum); IMP (Imipenemase Metallo-β-lactamase); SPM (Sao Paulo Metallo-β-lactamase); GIM (Germany Imipenemase); and VIM (Verona Integron-encoded Metallo-β-lactamase) [92].

#### AmpC

The intrinsic Class C β-lactamase, AmpC, is a chromosomally encoded cephalosporinase which drastically reduces sensitivity to penicillin’s and cephalosporins. High expression level of the *ampC* gene is commonly observed in resistant microbes, activated in part due to the presence of β-lactams, and its expression is controlled by the transcriptional regulator AmpR [110]. Carbapenems induce *ampC* expression, but their bactericidal activity and structural stability allow them to remain effective against AmpC+ strains [10]. Additionally, resistance to β-lactams can be attributed to mutations within AmpC repressor proteins, such as AmpD, which result in AmpC derepression [112]. Inhibition of penicillin binding prtoein 4 (PBP4) can act as an inducer of *ampC* expression, mutations to *ampR* and *dacB* (the gene that encodes PBP4) are commonly associated with *ampC* overexpression. This hyperproduction represents the primary mechanism underlying resistance to classic β-lactamase inhibitors, such as clavulanic acid and tazobactam. Even newer non-β-lactam inhibitors such as avibactam and relebactam become compromised when *ampC* is overexpressed [113, 114].

#### Extended spectrum β-lactamases (ESBL)

Extended Spectrum β-lactamases (ESBL) confer resistance to broad-spectrum cephalosporins. They have action across all Ambler classes, with the Extended Spectrum AmpC β-lactamase (ESAC) of class C being encoded on chromosomal *ampC* gene, the narrow-spectrum TEM and SHV β-lactamases along with the GES, VEB, PER, BEL-1, and CTX-M β-lactamases of class A and the Oxacillinases (OXA) of class D [10]. Within *P. aeruginosa*, class A and class D ESBLs are acquired via horizontal gene transfer, this is typically achieved via integrons (IMP and VIM). Such as in the case of *blaGES* gene which is inserted into the class 1 integron of a plasmid [111]. Of these ESBLs, acquiring single/double amino acid substitutions in the GES β-lactamases can confer resistance to carbapenems as well as reducing susceptibility to β-lactamase inhibitors [111, 112]. An XDR strain of *P. aeruginosa* (VIM–GES–CRPA) caused an epidemic in the United States in 2022 [115]. The outbreak strain exhibited resistance to cefepime, ceftazidime, piperacillin–tazobactam, aztreonam, carbapenems, ceftazidime–avibactam, and ceftolozane–tazobactam [115].

#### Carbapenemases

Carbapenemases are defined by their ability to hydrolyse carbapenems and are dispersed across Ambler classes A, B and D, with the class B MBLs being the most clinically prevalent. The MBLs degrade all β-lactams (except monobactams) and mostly associated with carbapenem resistance. In fact, only the Class C β-lactamases show low activity against carbapenems. Prevalent MBL carbapenemase families include NDM, IMP, and VIM [110]. Pan-resistant phenotypes can develop as a result of multiple resistance genes being harboured on the same plasmid as the MBL gene, as seen in a strain possessing both KPC-2 and VIM-2 genes alongside the aminoglycoside resistance gene *rmtD1* [102]. Of the SBL, notable examples include the KPC carbapenemases family (Class A) which can hydrolyse all β-lactams; and the OXA carbapenemases of Class D family. High-risk clones such as ST463 are shown to possess multiple carbapenemases (The class A KPC-2 and Class B AFM-1). Another Class A β-lactamase, PIB-1 contributes to imipenem tolerance [101]. Mutations can also affect sensitivity, with minor sequence variations significantly altering resistance levels. A single amino acid substitution distinguishes IMP-1 from IMP-6, with the latter showing greater resistance to meropenem. This can also be seen when observing the enhanced resistance to carbapenems posed by VIM-4, which has only two mutations making it distinct from VIM-1 [111].

In a case study, whole-genome sequencing of an XDR *P. aeruginosa* from corneal ulcer, identified the presence of the carbapenemase genes *blaVIM-80* and *blaGES-9* [4]. Similarly, investigations of ocular isolates belonging to sequence type ST308 revealed a class 1 integron carrying the acquired β-lactamase genes *blaTEM-1B*,* blaVIM-2*, and *blaPME-1*, which had not been previously reported in this lineage [91]. Consistent with these findings, analysis of 70 whole-genome-sequenced ocular isolates demonstrated that all the strains harboured a Pseudomonas-derived cephalosporinase (PDC) and at least one OXA-type β-lactamase, while only a small subset additionally carried other β-lactamases, including the MBL- VIM-80 and the class D enzyme LCR-1 [90].

#### Efflux pumps

There are several efflux pumps associated with carbapenem-resistance (MexAB-OprM, MexCD-OprJ, MexEF-OprN, and MexXY-OprM), all of which are regulated by several transcription factors - MexR, NalC, NalD, NfxB, NfxC, MexS, and MexZ [109]. Across several studies, carbapenem-resistant *P. aeruginosa *exhibited overexpression of efflux pumps, with most commonly related to MexAB-OprM pump [90, 116].

#### Biofilm

A study of keratitis isolates reported that all 34 strains harboured the calcium-binding kinase *ladS*, a key regulator that promotes biofilm formation [117]. In *P. aeruginosa*, the biofilm provides an additional layer of resistance resulting in recurring and/or chronic ocular infections [118, 119]. Alginate in the biofilm matrix contributes to stronger adhesion and antibiotic resistance [120]. The matrix impedes drug penetration and as the biofilm’s depth increases, microbial metabolic activity decreases, which makes the microbes less susceptible to the effect of antibiotics [118, 120]. The accumulation of β-lactamases present in the matrix helps hydrolyze β-lactam antibiotics such as imipenem and ceftazidime in a dynamic, spatially heterogenous process mediated by the bacteria [121]. A study from Iran reported that all biofilm-producing *P. aeruginosa* isolates from keratitis were 100% resistant to ceftazidime and carried *algD* gene, critical for the biosynthesis of alginate [122]. Similarly, an Indian study noted that strong biofilm-producing keratitis strains exhibited resistance to multiple antibiotics, including ceftazidime and cefepime [117]. In Australia, a keratitis isolate strain Paer17, which showed resistance to ticarcillin and aztreonam, also produced biofilm [81].

#### Mobile genetic elements (MGEs)

MGEs usually carry genes that either enable the microbe to persist within various ecological niches; provide antibiotic resistance or encode virulence determinants [12, 25]. In the case of β-lactamases, gene cassettes can become integrated to the genome via genetic recombination (*attI* and *attC*) mediated by an integrase enzyme [111]. A study found the most prevalent acquired β-lactamase genes were *blaPAO*,* blaOXA* in keratitis cases [90]. Ocular isolates sequence type ST308 was reported to harbour 24 acquired antimicrobial resistance genes including the *blaPAO* gene [91]. Another study identified the β-lactamase gene *blaLCR-1* in the flanking region of a class 1 integron, a theme shared in another study wherein the β-lactamase gene *blaNPS-1* was identified within a Tn3-like transposon harboring a class 1 integron [69, 123]. Whole genome sequencing of an Indian ocular isolate (strain VRFPA04) revealed the presence of several β-lactamase genes integrated into the chromosomal genome including *blaVIM-2*, *blaTEM-1B*, *blaPAO*, and *blaOXA-50*, of which *blaVIM-2* was encoded in a class 1 integron [124]. Another whole-genome–based study of keratitis isolates (*n* = 70) identified *blaPAO* and *blaOXA* as the most prevalent acquired resistance genes. The most frequent insertion sequences were ISPa1, ISPa6, and ISPa32, while the most common transposons were Tn4661, Tn6082, and Tn5563 [90].

### Polymyxin and mechanism of polymyxin resistance

Polymyxin is a naturally occurring antibiotic. It was first discovered in 1947 from *Bacillus polymyxa* [125] and later renamed as *Paenibacillus polymyxa*. Only polymyxin B and polymyxin E (colistin) are clinically significant out of the more than fifteen polymyxin variants that have been discovered so far [126–128]. Structurally, polymyxins consist of ten amino acids which form a heptapeptide ring attached to a tripeptide side chain and a fatty acid residue. They are positively charged cyclic lipopeptides that act mainly by damaging the outer membrane of Gram-negative bacteria. They interact with the negatively charged phosphate groups present in the lipid A portion of LPS on the bacterial outer membrane. After binding these sites, they displace the essential divalent cations such as magnesium (Mg²⁺) and calcium (Ca²⁺) [126]. The disruption eventually increases the permeability of the membrane, which causes cytoplasmic leakage and cell lysis [128, 129].

However, despite their potent activity against Gram-negative bacteria, resistance to polymyxins can arise through several mechanisms, including (i) lipid A modification, (ii) plasmid-borne resistance determinants, (iii) efflux pump overexpression, and (iv) adaptive and structural changes such as cross-resistance, heteroresistance, and biofilm formation [127].

#### Chemical modification of lipid A

*P. aeruginosa*, is chemical modification of lipid A. The addition of 4-amino-4-deoxy-L-arabinose (L-Ara4N) or phosphoethanolamine (pEtN) to the phosphate groups lead to lipid A modification [130]. The *arnBCADTEF* operon (also called *pmr* operon) mediates synthesis and attachment of L-Ara4N to lipid A, where enzyme such as EptA can add pEtN. These modifications greatly reduce the electrostatic attraction between polymyxin and the bacterial surface, lowering antibiotic susceptibility [127, 130].

Activation of lipid A-modifying pathways is controlled by multiple two-component regulatory systems (TCSs) such as PmrAB and PhoPQ. The membrane-bound sensor kinase PhoQ, a part of PhoPQ system, functions as a primary regulator [127, 131]. This kinase protein can sense environmental signals such as low Mg²⁺ and Ca²⁺, low pH, cationic antimicrobial peptides (CAMPs), and polymyxins. Mutation in the sensor kinase *phoQ* gene can activate this system, thereby conferring polymyxin resistance [132, 133]. Another membrane-bound sensor kinase system PmrAB contains a histidine residue that is essential for its activity. It becomes activated in response to environmental signals such as high levels of Fe³⁺ or low pH [134, 135]. This TCS is involved in upregulating the *arnBCADTEF* operon via an intermediary protein such as PmrD [136]. Polymyxin-resistant isolates are also frequently found to have mutations in the histidine kinase gene (*pmrB*), which constitutively activates this systems [127].

Moreover, the ParRS system functions as a central regulatory hub linking polymyxin resistance with broader antibiotic resistance pathways. Clinical studies have demonstrated that activation of ParRS system has been clinically linked to cross-resistance against several antibiotic classes, including polymyxins, aminoglycosides, fluoroquinolones, and β-lactams. This regulatory interaction contributes to the frequent emergence of multidrug-resistant *P. aeruginosa* and other Gram-negative pathogens [129, 137, 138]. Furthermore, the ColRS and CprRS systems also lead to polymyxin resistance in *P. aeruginosa*, especially under adaptive or clinically resistant conditions [129, 139].

One study reported that polymyxin resistance in MK *P. aeruginosa* isolates from India and Australia harbor the genes such as *pmrAB*,* phoPQ*,* cprRS*,* parRS*,* colRS* associated with regulation of the *arnBCADTEF* operon, but there was no direct relationship between gene presence and polymyxin resistance [140]. On the other hand, several single-nucleotide polymorphisms (SNPs) in chromosomally encoded genes were associated with polymyxin resistance among *P. aeruginosa* isolates in MK [140]. Strains with MICs to polymyxin of ≥ 256 µg/mL (very high resistance) had SNPs in several polymyxin-associated resistance genes including some combinations of *arnA* (Gln661Leu), *arnB* (Gln336Leu), *arnT* (Gly156Arg), *mipB* (Arg401del), *mpl* (Val358Ile or Ala303Val), *mprF* (Asn553Asp or Arg188His), *nalC* (Glu153Gln), *nalD* (Arg38Trp), *parR* (Ile93Thr), *pmrB* (Val6Ala), *speE2* (Ala3Val), *waaL* (Ala110Gly) [140].

#### Plasmid-borne resistance

In Gram-negative bacteria, plasmid-mediated resistance is considered as one of the most vital factors to contribute the global dissemination of polymyxin resistance. Previously, polymyxin resistance primarily occurred by chromosomal mutations. However, the identification of the mobilized colistin resistance *(mcr)* gene in *E. coli* in 2015 in China marked a major turning point in AMR research [141]. The *mcr* gene is located on a plasmid, which allows it to be rapidly transferred between different bacterial species through horizontal gene transfer. This rapid dissemination accelerates resistance far more quickly than traditional chromosomal mutations [142, 143]. Genes in the *mcr* family produce pEtN transferase enzymes that modify the lipid A region of the of LPS in the bacterial outer membrane. This certain modification lowers the membrane’s negative charge and diminishes the electrostatic binding between polymyxins and lipid A. As such, the binding affinity and antimicrobial activity of polymyxins are compromised [144, 145].

In contrast to chromosomal mechanisms, plasmid-borne *mcr* genes (*mcr*-1 to *mcr*-10) can spread horizontally via conjugative plasmids across diverse bacterial species, including *Salmonella enterica*, *P. aeruginosa*, and *Klebsiella pneumoniae* [143, 146–148]. Surveillance data from different studies indicate that the *mcr*-positive isolates have been associated with human, animal, and environmental samples in over 40 countries within 10 years of discovery. This shows how the gene might spread through food chains and clinical networks [130, 149]. However, *mcr* genes have not been found in keratitis isolates of *P. aeruginosa* [140].

#### Efflux pumps

Polymyxin resistance is significantly driven by efflux mechanisms and outer membrane remodelling, which together reduce the antibiotic’s ability to disrupt the bacterial cell envelope. In many bacterial pathogens, it has been shown that efflux pumps such AcrAB-TolC, MtrC-MtrD-MtrE, RosAB, KpnEF, and VexAB acquire resistance to polymyxins [127, 129]. Recent study suggests that polymyxin tolerance in *P. aeruginosa* is modulated by the MexXY-OprM efflux system [140]. The regulatory genes *nalC* and *nalD* control the expression and activity of this efflux pump and SNPs in these genes were associated with resistance to polymyxin B and colistin [140].

#### Physiological and adaptive processes

In addition to genetic determinants, polymyxin resistance may be driven by physiological and adaptive processes such as cross-resistance, heteroresistance, and biofilm formation. Cross-resistance typically arises when a single molecular mechanism mediates resistance across multiple antibiotic classes, allowing bacteria to tolerate a broad spectrum of antimicrobial agents. This process occurs mostly due to the overexpression of efflux pumps and the activation of two-component regulatory systems [150, 151]. Upregulation of the MexAB-OprM efflux pump, which is associated with fluoroquinolone and β-lactam resistance, also contributes to reduced susceptibility to polymyxins [152]. Heteroresistance refers where both polymyxin-susceptible and polymyxin-resistant cells coexist within one bacterial population. This condition frequently arises from temporary mutations in regulatory genes like *pmrB* or *phoQ*, leading to reversible alterations in lipid A. The resistant subpopulations can revert to susceptibility, when antibiotic pressure is removed. This phenomenon demonstrates a bacterial dynamic adaptation to environmental stress [130, 150, 151]. Biofilm is another mechanism that further increases polymyxin resistance by establishing protective environments where antibiotic diffusion is restricted. The spatial heterogeneity of biofilms creates microenvironments where polymyxins are neutralized at the periphery, thereby allowing chronic persistence [78, 141]. A recent investigation on colistin-resistant *P. aeruginosa* revealed that all resistant isolates produced biofilms and exhibited alterations in efflux-pump and porin genes, indicating a correlation between biofilm production and polymyxin resistance [153].

## Antibiotic resistance data of *P. aeruginosa* in keratitis in different continents

In this study, literature on antibiotics resistance in *P. aeruginosa* on keratitis was searched in PubMed, MEDLINE, Web of Science, Scopus, Google scholar. The search was performed using a wide spectrum of terms related with antimicrobial resistance in *P. aeruginosa* in ocular infections including antibiotic susceptibility, antimicrobial susceptibility, antimicrobial resistance, antibiotic resistance, *Pseudomonas aeruginosa*, Gram-negative bacteria, microbial keratitis, infectious keratitis, bacterial keratitis, corneal ulcer, surveillance study, and ARMOR study. Reference lists of all the related literature were further manually checked for relevant information and found potential articles that may have been included in our initial search. Then, all the collected data were used to describe the antibiotics resistance in *P. aeruginosa* in MK (Table 1).

The data demonstrate that the antibiotic resistance patterns of *P. aeruginosa* isolates from keratitis patients vary significantly by region (Table 1). The Asian studies showed a high level of resistance to fluoroquinolones and third-generation cephalosporin. In India, fluoroquinolone resistance has been shown to range from 2.8% [54] to 100% resistance [154, 155], with resistance to moxifloxacin and ciprofloxacin having rates between 40% and 100% in different study periods [155–159]. Ceftazidime and cefazolin resistance rates were the highest among the third-generation cephalosporins, ranging from 60% to 100% in majority of the cohorts [63, 155, 157, 158, 160]. The resistance rate of aminoglycoside varied throughout the studies, where amikacin, gentamicin, and tobramycin showed resistance rates as high as 100% in some investigations [155]. Moreover, most of the research showed a moderate aminoglycoside resistance incidence, accounting for 33.3% to 64.6% [63, 156, 158, 159, 161]. Chloramphenicol resistance rate was observed in the Indian isolates, ranging from 45.45% to 100% [117, 154, 155, 157, 162]. High resistance to tigecycline (100%), ticarcillin/clavulanic acid (91%) and piperacillin/tazobactam (65%) were reported in India [117]. Earlier data from India [162] showed a substantial resistance rate to ticarcillin (63.64%) and trimethoprim–sulfamethoxazole (72.73%). A Pakistani study [163] showed very high resistance to amoxicillin (97.4%), cephradine (92.3%) and chloramphenicol (71.7%) but lower rates for ciprofloxacin (18%) and imipenem (15.6%) (Table 1).

A multicountry dataset encompassing Singapore, Hong Kong, Japan, South Korea, Thailand, the Philippines, Taiwan, and China showed resistance rates across several antibiotic classes, which ranges from 6% to 17% [164]. Overall, this regional analysis revealed comparatively lower resistance levels than those reported in India, highlighting potential geographic or clinical differences in antimicrobial use. A Chinese investigation further supported this trend, documenting a markedly reduced ciprofloxacin resistance rate of approximately 10% within a small cohort (*n* = 10) [165]. In sharp contrast, isolates from Indonesia exhibited a far more alarming profile, showing complete resistance to several β-lactam antibiotics (penicillins and cephalosporins), as well as moxifloxacin, ertapenem, chloramphenicol, tetracycline, and co-trimoxazole. Resistance to other antibiotics, including ciprofloxacin, levofloxacin, gentamicin, amikacin, and imipenem varied from 6.52% to 26.09% [166]. Studies from Iran reported almost 100% resistance to vancomycin, cefazolin, chloramphenicol, and trimethoprim–sulfamethoxazole, whereas resistance to fluoroquinolones and aminoglycosides remained low (0% − 7%) [167–169] (Table 1).

In Europe, resistance levels were generally lower than those recorded from Asia. In Portugal, cotrimoxazole resistance was 27.91% in one research [170] and 44.4% in another [171], although fluoroquinolone and aminoglycoside resistance was low (4.65%) in both studies. Isolates from Italy exhibited 100% resistance to trimethoprim-sulfamethoxazole and many β-lactam antibiotics (ampicillin, cefotaxime, and ceftriaxone), but they were completely susceptible to ceftazidime, cefepime, ciprofloxacin, levofloxacin, gentamicin, amikacin, and tobramycin [3]. In Spain, data indicated low resistance ratio to fluoroquinolones (4.35–5.9%), aminoglycosides (0%-7.9%), ceftazidime (5.3-13.04%) and colistin (0%) [172, 173]. In contrast, investigation by another group found 100% resistance to erythromycin [172].

Compared to Asian countries, in North America, particularly in the United States resistance levels were generally low against several important antibiotic classes [174, 175]. Resistance to aminoglycosides like gentamicin and tobramycin, fluoroquinolones like ciprofloxacin and moxifloxacin, and ceftazidime stayed below 7%. However, in a study high resistance to ampicillin, tetracycline, trimethoprim, and sulfasoxazole was observed which ranges from 94% to 100% [174]. Another study documented low resistance to fluoroquinolones (1.95%–3.9%), aminoglycosides (0.65%), and polymyxin (0%), but total resistance to erythromycin and trimethoprim [176]. It is noteworthy that isolates with *blaVIM* and *blaGES* genes showed widespread multidrug resistance, including 100% resistance to the majority of tested antibiotics [177] (Table 1).

In Australia, variable patterns of antibiotic resistance among *P. aeruginosa* keratitis isolates have been reported. One study documented high resistance rates to imipenem (78.6%), ceftazidime (57.14%), and ciprofloxacin (50%), whereas resistance to other antibiotic classes, including gentamicin, tobramycin, and polymyxin B, remained low at 0%, 7.14%, and 7.14%, respectively [63]. In contrast, another study reported lower resistance rates to aminoglycosides (13.04%) and fluoroquinolones (10.87–17.39%) and very low rates for netilmicin (2.17%), aztreonam (2.17%) and ticarcillin (4.35%) [178]. Additionally, complete susceptibility to ciprofloxacin, ofloxacin, gentamicin, and combination therapies such as cefazolin plus gentamicin and chloramphenicol plus gentamicin has also been reported in Australian isolates, while high resistance was observed against moxifloxacin, chloramphenicol, and cefazolin [179] (Table 1).

Egypt is the principal representative of African studies. In contrast to reports of complete susceptibility to amikacin and gatifloxacin, high resistance rates (80–100%) have been documented for several antibiotics, including ceftazidime, cefotaxime, ceftriaxone, chloramphenicol, azithromycin, and tetracycline [149]. Similarly, another study from Egypt reported substantial resistance to multiple antibiotic classes, with high resistance observed for tobramycin (73.5%), ciprofloxacin (65.3%), gentamicin (61.2%), amikacin (59.2%), chloramphenicol (55.3%), and meropenem (51%) among *P. aeruginosa* keratitis isolates [150]. In contrast, resistance to antibiotics used as a last resort such as colistin, gatifloxacin, and polymyxin B remained relatively low, which stood at 10.2%, 16.3%, and 18.4% respectively (Table 1).

**Table 1:** Antibiotic resistance patterns of *P. aeruginosa* isolates from keratitis patients across different continents

Continent	Country	Antibiotics	Sample no.	References
Asia	India	Moxifloxacin (100%) Chloramphenicol (100%) Ofloxacin (71.4%) Ceftazidime (71.4%)	8	[154]
	India	Gentamicin (63.6%) Cefazolin (100%) Ciprofloxacin (100%) Chloramphenicol (90.9%) Amikacin (9.1%) Norfloxacin (77.2%)	22	[157]
	India	Ofloxacin (13.1%) Fluoroquinolones (13.1%) Gentamicin (13.5%) Ceftazidime (5.5%)	887	[180]
	China	Ciprofloxacin (10%)	10	[165]
	Singapore, India, Hong Kong, Japan, South Korea, Thailand, Philippines, Taiwan, China	Cefepime (11.6%) Amikacin (13.2%) Imipenem (13.8%) Ceftazidime (14.7%) Piperacillin/Tazobactam (15%) Tobramycin (15%) Besifloxacin (15.4%) Gentamicin (15.4%) Levofloxacin (15.4%) Gatifloxacin (16.3%) Moxifloxacin (16.9%) Ciprofloxacin (17.6%) Polymyxin B (6%)	319	[164]
	India	Levofloxacin (85%) Ceftazidime (70.9%) Imipenem (58.33%) Amikacin (58.33%) Gentamicin (63.64%) Ciprofloxacin (72.73%) Colistin (10.71%)	34	[161]
	India	Colistin (3%, *n* = 37) Ofloxacin (33%, *n* = 15) Tobramycin (35%, *n* = 37) Amikacin (37%, *n* = 43) Ciprofloxacin (43%, *n* = 42) Pipercillin (49%, *n* = 37) Gatifloxacin (50%, *n* = 38) Moxifloxacin (53%, *n* = 38) Ceftazidime (71%, *n* = 41)	15–43	[159]
	India	Amikacin (10.3%) Chloramphenicol (59.8%) Ciprofloxacin (17.1%) Gentamicin (10.3%) Gatifloxacin (26.5%) Ofloxacin (26.5%) Tobramycin (26.5%) Moxifloxacin (17.7%)	117	[181]
	India	Amikacin (39.2%) Moxifloxacin (47.2%) Ciprofloxacin (43.2%) Gentamicin (40.3%) Tobramycin (47.7%)	176	[156]
	India	Ciprofloxacin (45.45%) Levofloxacin (18.18%) Ticarcillin (63.64%) Chloramphenicol (45.45%) Aztreonam (0%) Piperacillin-tazobactam (0%) Meropenem (0%) Ceftazidime (0%) Gentamicin (45.45%) Amikacin (0%) Cefotaxime (9.09%) Cotrimoxazole (72.73%)	11	[162]
	India	Ciprofloxacin (8.4%, *n* = 95) Chloramphenicol (86.7%, *n* = 15) Cefazolin (100%, *n* = 15) Gentamicin (46.7%, *n* = 15) Norfloxacin (53.3%, *n* = 15)	95/15	[160]
	India	Moxifloxacin (31.58%, *n* = 19) Gatifloxacin (21.05%, *n* = 19) Tobramycin (38.46%, *n* = 26) Cefazolin (65.38%, *n* = 26)	19/26	[182]
	Indonesia	Meropenem (4.35%) Amikacin (6.52%) Imipenem (10.87%) Piperacillin-tazobactam (15.22%) Ciprofloxacin (21.74%) Gentamicin (23.91%) Aztreonam (28.26%) Levofloxacin (26.09%) Amoxicillin-clavulanic acid (100%) Ampicillin (100%) Ampicillin-sulbactam (100%) Cefazolin (100%) Cefotaxime (100%) Cefoxitin (100%) Ceftriaxone (100%) Chloramphenicol (100%) Ertapenem (100%) Tetracycline (100%) Cotrimoxazole (100%)	38	[166]
	Pakistan	Amoxicillin (97.4%) Cephradine (92.3%) Neomycin (30.8%) Ciprofloxacin (18%) Chloramphenicol (71.7%) Imipenem (15.6%)	39	[163]
	India	Cefazolin (100%) Chloramphenicol (100%) Gentamicin (100%) Tobramycin (100%) Amikacin (100%) Ceftazidime (100%) Ciprofloxacin (100%) Ofloxacin (100%) Gatifloxacin (100%) Moxifloxacin (100%) Imipenem (0%) Colistin (0%)	12	[155]
	Iran	Amikacin (3%) Cefazolin (100%) Chloramphenicol (97%) Gentamicin (7%) Imipenem (4%) Tetracycline (71%) Trimethoprim (96%) Vancomycin (100%) Ceftazidime (0%) Ciprofloxacin (0%)	52	[167]
	India	Ciprofloxacin (75%) Levofloxacin (50%) Gentamicin (33.33%) Tobramycin (50%) Piperacillin (41.67%) Imipenem (58.33%) Ceftazidime (50%) Polymyxin B (25%)	12	[63]
	India	Gatifloxacin (40%) Ciprofloxacin (44%) Moxifloxacin (49%) Tobramycin (49%) Amikacin (56%) Ceftazidime (61%) Cefuroxime (63%) Cefazolin (68%) Tetracycline (65%) Polymyxin B (0%)	57	[158]
	Iran	Vancomycin (100%) Cefazolin (100%) Chloramphenicol (100%) Trimethoprim–sulfamethoxazole (100%) Gentamicin (3.5%) Amikacin (0%) Imipenem (0%) Ciprofloxacin (0%) Ofloxacin (0%) Norfloxacin (0%)	57	[168]
	India	Chloramphenicol (94%) Ciprofloxacin (30%) Moxifloxacin (88%) Gatifloxacin (21%) Ofloxacin (21%) Levofloxacin (38%) Gentamicin (18%) Amikacin (15%) Tobramycin (18%) Ceftazidime (38%) Cefepime (33%) Imipenem (15%) Doripenem (21%) Meropenem (15%) Piperacillin/Tazobactam (65%) Ticarcillin/Clavulanic Acid (91%) Cefoperazone/Sublactam (68%) Tigecycline (100%) Colistin (0%)	34	[117]
	India	Ciprofloxacin (2.8%)	106	[54]
	Iran	Vancomycin (100%) Cefazolin (100%) Chloramphenicol (100%) Trimethoprim–sulfamethoxazole (100%) Gentamicin (3.5%) Amikacin (0%) Imipenem (0%) Ciprofloxacin (0%) Ofloxacin (0%) Norfloxacin (0%)	57	[169]
Europe	Portugal	Fluoroquinolones (4.65%) Aminoglycosides (4.65%) Cotrimoxazole (27.91%)	43	[170]
	Italy	Ampicillin (100%) Ampicillin–sulbactam (100%) Trimethoprim–sulfamethoxazole (100%) Amoxicillin–clavulanic acid (100%) Aztreonam (25%) Cefotaxime (100%) Ceftriaxone (100%) Imipenem (12.5%) Ceftazidime (0%) Cefepime (0%) Piperacillin (0%) Amikacin (0%) Gentamicin (0%) Tobramycin (0%) Ciprofloxacin (0%) Levofloxacin (0%)	8	[3]
	Portugal	Trimethoprim/sulfamethoxazole (44.4%)	9	[171]
	Spain	Ceftazidime (13.04%) Fluoroquinolones (4.35%) Erythromycin (100%) Aminoglycosides (0%)	23	[172]
	Spain	Ceftazidime (5.3%, *n* = 38) Cefepime (5.3%, *n* = 38) Imipenem (5.3%, *n* = 38) Aztreonam (2.9%, *n* = 34) Levofloxacin (5.9%, *n* = 34) Ciprofloxacin (5.3%, *n* = 38) Gentamicin (7.9%, *n* = 38) Amikacin (0%, *n* = 38) Colistin (0%, *n* = 35%) Meropenem (0%, *n* = 38)	34/38	[173]
North America	USA	Ampicillin (100%) Gentamicin (6%) Neomycin (44%) Ceftazidime (2%) Polymyxin B (2%) Trimethoprim (100%) Tetracycline (100%) Sulfasoxazole (94%) Tobramycin (6%) Moxifloxacin (3%) Ciprofloxacin (2%)	57	[174]
	USA	Tobramycin (2%) Amikacin (2%) Ciprofloxacin (6%) Gentamicin (5%) Ceftazidime (0%)	82	[175]
	USA	Erythromycin (100%) Trimethoprim (100%) Moxifloxacin (1.95%) Levofloxacin (3.9%) Tobramycin (0.65%) Polymyxin B/trimethoprim (0%) Polymyxin B (0%) Rifampin (0%)	154	[176]
	USA	Amikacin (100%) Cefepime (100%) Ceftazidime (100%) Ciprofloxacin (100%) Gentamicin (100%) Levofloxacin (100%) Meropenem (100%) Tobramycin (100%)	9	[177]
Australia	Australia	Ciprofloxacin (50%) Levofloxacin (14.29%) Gentamicin (0%) Tobramycin (7.14%) Piperacillin (21.43%) Imipenem (78.57%) Ceftazidime (57.14%) Polymyxin B (7.14%)	14	[63]
	Australia	Ticarcillin (4.35%) Piperacillin (8.7%) Ceftazidime (13.04%) Aztreonam (2.17%) Tobramycin (13.04%) Netilmicin (2.17%) Norfloxacin (10.87%) Ciprofloxacin (10.87%) Ofloxacin (17.39%) Moxifloxacin (17.39%)	46	[178]
	Australia	Cefalotin/Cefazolin (100%) Chloramphenicol (100%) Ciprofloxacin/Ofloxacin (0%) Gentamicin (0%) Cefalotin/Cefazolin + Gentamicin (0%) Chloramphenicol + Gentamicin (0%)	50	[179]
Africa	Egypt	Azithromycin (100%) Ceftazidime (83.3%) Ceftriaxone (80%) Chloramphenicol (83.3%) Ciprofloxacin (50%) Cefotaxime (100%) Gentamicin (36.4%) Levofloxacin (21.2%) Moxifloxacin (33.3%) Ofloxacin (42.9%) Tetracycline (100%) Tobramycin (44.4) Amikacin (0%) Gatifloxacin (0%)	13	[183]
	Egypt	Tobramycin (73.5%) Ciprofloxacin (65.3%) Gentamicin (61.2%) Amikacin (59.2%) Chloramphenicol (55.3%) Meropenem (51%) Ofloxacin (49%) Levofloxacin (46.9%) Cefazolin (42.9%) Imipenem (38.8%) Ceftazidime (38.8%) Azithromycin (36.7%) Piperacillin-Tazobactam (32.7%) Ceftazidime-Avibactam (22.4%) Polymyxin B (18.4%) Gatifloxacin (16.3%) Colistin (10.2%)	49	[184]

## Summary and research gap in the literature

*P. aeruginosa* keratitis isolates deploy a multitude of overlapping resistance mechanisms. For fluoroquinolones, resistance typically involves mutations in the *gyrA* and *parC*, overexpression of efflux pumps due to mutations in their regulators, and acquisition of plasmid-borne modifiers (Qnr or CrpP) enzymes [57, 63]. Aminoglycoside resistance similarly arises from AME enzymes (AAC, ANT, APH families) and 16 S rRNA methyltransferases, in concert with reduced uptake and MexXY-OprM efflux [57, 63]. β-Lactam resistance is likewise multifactorial: derepression or mutation of chromosomal AmpC β-lactamase, upregulated efflux, along with acquisition of horizontally transferred β-lactamases [90] whereas resistance to polymyxins is associated with lipid A modification in the outer membrane, efflux pump overexpression, cross-resistance, and heteroresistance [130, 140, 150–152]. Biofilm mediated resistance is associated with all different groups of antibiotics. The resistance data showed substantial geographic variation, with high resistance in India and parts of Southeast Asia, moderate resistance in Egypt and Australia, and generally low resistance in Europe and North America. 

In *P. aeruginosa* keratitis, severity is largely determined by two distinct pathogenic groups: cytotoxic and invasive strains. Strains expressing ExoS are classified as invasive, as they invade and persist within epithelial cells [5], whereas ExoU-positive strains are cytotoxic and rapidly induce host cell death due to the potent phospholipase activity of ExoU [6].

Although this virulence-based dichotomy is well-characterized, comparative studies examining antibiotic resistance between cytotoxic and invasive keratitis isolates remain limited. For instance, an Australian study in 2008 reported that *exoU*-positive strains exhibited higher resistance to fluoroquinolones [81]. Consistently, another study found that all but one *exoU* strain were resistant to at least two of the three tested fluoroquinolones, and all were resistant to three or more β-lactams, except for one strain, which was resistant only to ticarcillin and imipenem [56]. A more recent study comparing *exoU* and *exoS* keratitis isolates demonstrated that *exoU* strains were more resistant to fluoroquinolones (ciprofloxacin, levofloxacin), and aminoglycosides (gentamicin, and tobramycin) [57].

Future research should focus on strain-specific resistance patterns to better understand the association between genotype and antimicrobial resistance. Continuous, region-specific surveillance of *P. aeruginosa* isolates from keratitis patients is essential to monitor emerging resistance trends. Such surveillance will facilitate timely detection of shifts in resistance, guide empiric therapy, and inform public health strategies to prevent the spread of multidrug-resistant strains. In parallel, studies should explore the relationship between antibiotic resistance and virulence factors, including ExoU and ExoS, to determine association with resistance and disease severity. Therapeutically, efforts should focus on combination antibiotic strategies (e.g., antibiotic plus aminoglycoside, dual β-lactam), antimicrobial peptides, non-antibiotic approaches such as anti-virulence drugs, bacteriophage therapy, or host-directed treatments, and the development of region-specific guidelines for empiric management of *P. aeruginosa* keratitis.

## Conclusion

*P. aeruginosa* keratitis isolates show resistance to antibiotics using complex intrinsic and acquired resistance mechanisms and resistance profile varies across the different regions. Continuous, region-specific surveillance of antimicrobial resistance focusing on cytotoxic and invasive strains will be critical for informing more effective and targeted therapeutic interventions to prevent vision-loss.

## Data Availability

Data is provided within the manuscript.
